# An *ex vivo*, assessor blind, randomised, parallel group, comparative efficacy trial of the ovicidal activity of three pediculicides after a single application - melaleuca oil and lavender oil, eucalyptus oil and lemon tea tree oil, and a "suffocation" pediculicide

**DOI:** 10.1186/1471-5945-11-14

**Published:** 2011-08-24

**Authors:** Stephen C Barker, Phillip M Altman

**Affiliations:** 1 Parasitology Section, School of Chemistry & Molecular Biosciences, and UniQuest Pty Ltd, University of Queensland, Brisbane, QLD, Australia 4072; 2 Altman Biomedical Consulting Pty. Ltd. 20 Folly Point, Cammeray, New South Wales, Australia

## Abstract

**Background:**

There are two components to the clinical efficacy of pediculicides: (i) efficacy against the crawling-stages (lousicidal efficacy); and (ii) efficacy against the eggs (ovicidal efficacy). Lousicidal efficacy and ovicidal efficacy are confounded in clinical trials. Here we report on a trial that was specially designed to rank the clinical ovicidal efficacy of pediculicides. Eggs were collected, pre-treatment and post-treatment, from subjects with different types of hair, different coloured hair and hair of different length.

**Method:**

Subjects with at least 20 live eggs of *Pediculus capitis *(head lice) were randomised to one of three treatment-groups: a melaleuca oil (commonly called tea tree oil) and lavender oil pediculicide (TTO/LO); a eucalyptus oil and lemon tea tree oil pediculicide (EO/LTTO); or a "suffocation" pediculicide. Pre-treatment: 10 to 22 live eggs were taken from the head by cutting the single hair with the live egg attached, before the treatment (total of 1,062 eggs). Treatment*: *The subjects then received a single treatment of one of the three pediculicides, according to the manufacturers' instructions. Post-treatment: 10 to 41 treated live eggs were taken from the head by cutting the single hair with the egg attached (total of 1,183 eggs). Eggs were incubated for 14 days. The proportion of eggs that had hatched after 14 days in the pre-treatment group was compared with the proportion of eggs that hatched in the post-treatment group. The primary outcome measure was *% ovicidal efficacy *for each of the three pediculicides.

**Results:**

722 subjects were examined for the presence of eggs of head lice. 92 of these subjects were recruited and randomly assigned to: the "suffocation" pediculicide (n = 31); the melaleuca oil and lavender oil pediculicide (n = 31); and the eucalyptus oil and lemon tea tree oil pediculicide (n = 30 subjects). The group treated with eucalyptus oil and lemon tea tree oil had an *ovicidal efficacy *of 3.3% (SD 16%) whereas the group treated with melaleuca oil and lavender oil had an *ovicidal efficacy *of 44.4% (SD 23%) and the group treated with the "suffocation" pediculicide had an *ovicidal efficacy *of 68.3% (SD 38%).

**Conclusion:**

Ovicidal efficacy varied substantially among treatments, from 3.3% to 68.3%. The "suffocation" pediculicide and the melaleuca oil and lavender oil pediculicide (TTO/LO) were significantly more ovicidal than eucalyptus oil and lemon tea tree oil pediculicide (EO/LTTO) (P < 0.0001). Ranking: 1. "Suffocation" pediculicide (68.3% efficacy against eggs); 2. Melaleuca oil and lavender oil (44.4%) pediculicide; 3. Eucalyptus oil and lemon tea tree oil (3.3%) pediculicide. The "suffocation" pediculicide and TTO/LO are also highly efficacious against the crawling-stages. Thus, the "suffocation" pediculicide and TTO/LO should be recommended as first line treatments.

**Trial Registration:**

The study was listed at the Australian/New Zealand Clinical Trial Registry (ANZCTR): reg. no. 12609000884202.

## Background

Pediculosis, infection with head lice, *Pediculus capitis*, is common in children of elementary-school age. There are two components to the clinical efficacy of pediculicides. First, efficacy against the crawling-stages (the three nymphal instars n1, n2, n3, and the adults), hereafter called lousicidal efficacy. And second, efficacy against the eggs in the hair, hereafter called ovicidal efficacy. Lousicidal efficacy and ovicidal efficacy are, perforce, confounded in *in vivo *clinical trials where both the lice and eggs are treated at the same time on the scalp and the end-point is defined as 'louse-free post-treatment'. For example, subjects may be louse-free after two treatments, one week apart, regardless of the ovicidal efficacy of the pediculicide so long as the pediculicide kills all of the crawling-stages present in the hair at the start of the trial (first treatment) and then kills all of the crawling-stages that hatch between the treatments, at the second treatment. In the last 10 years or so two new types of pediculicides have found wide acceptance in many countries: essential oil pediculicides and pediculicides designed to "suffocate" head lice and their eggs.

The *in vivo *efficacy of the "suffocation" pediculicide, and the melaleuca oil and lavender oil pediculicide, that we studied in the trial reported here, were studied in a previous trial reported in this journal [1]. 98% of subjects treated with the "suffocation" pediculicide, and 98% of subjects treated with the melaleuca oil and lavender oil pediculicide, were louse-free at the end of that 14 day trial [1]. It was not known, however, how ovicidal these two pediculicides were; the trial reported here was designed to assess the ovicidal activity of these pediculicides, and to compare their ovicidal efficacy with another essential oil pediculicide (eucalyptus oil and lemon tea tree oil (EO/LTTO)) that is marketed with the claim of 'kills eggs.' This claim has been approved by the Therapeutic Goods Administration (TGA) of the Australian Government. Ovicidal efficacy may be assessed *in vitro: *when eggs are collected from an infected subject and then immersed in pediculicides in the laboratory for a period of time, after which they are washed and then incubated for 14 days. The limitation of such *in vitro *studies is that the eggs are not treated *in vivo *(on the head) and that the pediculicide is not washed out *in vivo*. Thus, the exposure of the eggs to the pediculicide *in vitro *is different to the exposure on the head (*in vivo*), and thus, *in vitro *results may or may not indicate the efficacy of the pediculicides when used by health professionals and parents *in vivo*. *In vivo *studies of ovicidal efficacy are confounded by the difficulty, often impossibility, of tracking individual eggs on a head from pre-treatment to 14 days post-treatment. Dodd [2] reviewed studies of the *ex vivo *efficacy of pediculicides [3-5] and then recommended measuring ovicidal efficacy, by comparing the hatch-rate of eggs collected pre-treatment with the hatch-rate of eggs collected post-treatment. Comparing the proportion of eggs that hatch pre-treatment and post-treatment on the same individual allows variation in hatchability among subjects to be taken into account. The proportion of eggs that hatch among heads varies; this variation is not well understood but may be related to the presence/absence of sufficient males on a subject to fertilize all females and inherent variation in the "quality" of eggs laid by different female lice.

Here we report on a clinical trial that was specially designed to determine the *ex vivo *clinical ovicidal efficacy of pediculicides. A "league table" of the clinical ovicidal efficacy and, for that matter, the clinical lousicidal efficacy of the pediculicides that are sold in particular markets would be of interest to both consumers and health professionals.

## Methods

### Objectives and interventions

To compare, *ex vivo*, the ovicidal efficacy of three pediculicides after a single application on subjects with live head lice eggs:

1. Pediculicide containing melaleuca oil (commonly called tea tree oil) 10% w/v and lavender oil 1%w/v (TTO/LO) (NeutraLice Natural Lotion^® ^Key Pharmaceuticals Pty Ltd, Australia) presented as a clear oily solution

2. "Suffocation" pediculicide containing benzyl alcohol, mineral oil, polysorbate 80, sorbitan monooleate, Carbopol 934, water and triethanolamine (NeutraLice Advance^® ^Key Pharmaceuticals Pty Ltd, Australia) presented as a white opaque lotion.

3. Pediculicide containing eucalyptus oil 11% w/w and lemon tea tree oil 1% w/w (EO/LTTO) (MOOV Head Lice Solution^®^, Ego Pharmaceuticals Pty Ltd, Australia) presented as a clear oily solution

### Methodology

This was an assessor-blind, randomised, parallel group, comparative study. The study population was elementary school-aged children (aged 4 yrs to 12 yrs) in Queensland, Australia, with live eggs of head lice in their hair. After written, informed consent had been provided, subjects were screened by visual inspection for the presence of putatively live eggs of head lice in their hair. The hair was divided into six sections. Each section of hair was examined for the presence of putatively live eggs by an expert technician - a magnifying glass was not needed. Eggs were classified as putatively alive if they were attached to a hair shaft within about 1 cm of the scalp and if they were not black (dead) nor white to translucent (hatched). Putatively live eggs were counted with a hand-operated click-counter. Subjects with at least 20 putatively live eggs in their hair were randomised to one of the three treatment groups. Subjects with scalp disease, or a history of allergies, and those who were treated with a pediculicide, or hair dye or bleach in the four weeks prior to the trial, were excluded from the trial.

#### Criterion for a live egg, a dead egg and hatched egg

Each egg was examined individually, within 2 hrs of collection of being removed from the head, with a stereo light microscope of 16 power to check for eggs that looked like they were alive with the naked eye, but were in fact were dead or had hatched. None of the eggs that were classified as alive by their appearance and close proximity to the scalp (within 1 cm) were found to be either dead or had hatched when examined with a microscopy.

*Live egg*: the egg was less than about 1 cm from the scalp; and the operculum was closed; and the egg had a uniform ovoid shape; and the egg had a uniform density and appearance (may have had an "eye spot" but this depended on the age of the egg. (Refer to Sonnberg et al for some excellent colour pictures of eggs of *P. capitis *[6])).

*Dead egg: *the egg was misshapen, shrivelled, indented or irregular in shape; or the egg had a non-uniform density with parts of the egg clear whereas other parts of the egg were opaque.

*Hatched egg*: operculum was open and nymph was not in the egg.

#### Collection, transport and incubation of eggs

Hair shafts with eggs attached less than 1 cm from the scalp were cut with hairdresser's scissors. The hair shaft was then put in a plastic tube (Corning 50 mL Centrifuge Tubes; CentriStar™ Cap-Polypropylene-Sterile). All of the pre-treatment hair shafts from one subject were put into one plastic tube whereas the hair shafts collected post-treatment were all put into a separate tube. The tubes were sealed with screw caps, put in a polystyrene container to minimize temperature variation, and taken to the laboratory. Then the pre-treatment and post-treatment eggs were counted and placed in separate Petri dishes in an incubator at 29 to 30°C and 70% relative humidity. Eggs were put into the incubator within 4 hrs of collection. Eggs were held in the incubator for 14 days.

#### Randomisation and blinding

Eligible subjects were randomly assigned to be treated with one of the three pediculicides, by a computer generated code with blocked randomisation (groups of six). This trial was assessor-blind. The person applying the treatment could not be blinded because the three pediculicides were easily identifiable by their physical attributes and "feel"; however, the person (MA) who classified eggs as either alive, dead or hatched was blinded to the treatment. Analysts were blinded to the treatment group until after the analyses.

#### Treatments and criteria for evaluation of efficacy

Enrolled subjects were treated once with one of the three pediculicides, according to the manufacturers' instructions. The louse-combing procedure normally used in combination with the TTO/LO and the "suffocation" pediculicide would have confounded our study, so it was not done. After the treatment, at least l0 live eggs, but more eggs if more than 10 eggs were present, were taken from the hair of each enrolled subject, as for the pre-treatment eggs. The pre-treatment and post-treatment eggs were examined again with the microscope on Day 14 to determine whether the eggs had hatched, partly hatched or had not hatched at all. Partly hatched eggs were classified as unhatched eggs; in these cases the partly hatched nymph was always dead. The number of hatched (empty) and unhatched eggs was counted on Day 14. The proportion of eggs that had hatched after 14 days was compared in the pre-treatment and post-treatment groups of eggs. The primary outcome measure was *percent *(%) *ovicidal efficacy *for each treatment-group (1 - [Post-Treatment Hatching Rate/Pre-Treatment Hatching Rate] × 100%). *Hatch-Rate = *number of eggs that had hatched divided by the number of live eggs that had been collected. Characteristics of the subjects and their hair were recorded: hair type (whether curly, straight or wavy), hair colour (whether black, blonde, brown, red), hair length (cm), subject gender and school attended (subjects attended three different schools in western Brisbane). Grade at school was a surrogate for subject age. Subjects were from eight school grades: "preparation year" (ca. 5 years) and grades 1 (ca. 6 years), 2 (ca. 7 years), 3 (ca. 8 years), 4 (ca. 9 years), 5 (ca. 10 years), 6 (ca. 11 years), and 7 (ca. 12 years).

#### Dosage and dosage regimen

The dose and method of application was that recommended by the manufacturers: all three pediculicides were applied for 10 minutes. After the EO/LTTO and the TTO/LO were applied, the hair was covered with a plastic ("shower") cap as per the manufacturers instructions. The EO/LTTO was washed from the hair with regular shampoo whereas the TTO/LO and the "suffocation" pediculicide were washed from the hair with tap-water. Then the hair was dried with a towel.

#### Criteria for evaluation of safety (tolerance)

Safety was evaluated by the incidence and severity of Adverse Events (AEs), and the likelihood in the opinion of the Investigator (SCB), that those AEs were caused by the pediculicides. The scalps of subjects were examined after the treatments and subjects were asked "was the hair treatment okay?" to elicit responses. The incidence and severity of adverse events was compared among treatment groups. Fisher's Exact Test was used to compare the proportions of subjects who reported an AE among the three treatment groups.

#### Statistical analyses

In our statistical analyses, the egg was the investigative unit whereas whether the egg hatched or not was the outcome. We used a framework of Generalised Estimating Equations (GEE) so that we could take into account the: (i) different numbers of eggs collected, pre-treatment (10 to 22 eggs) and post-treatment (10 to 41 eggs), from the 92 subjects; (ii) the three hair types; (iii) the four hair colours (iv) hair length (in cm); (v) the eight different age classes; (vi) gender; and (vii) the three different schools attended (populations). Logistic regression models were fitted to the data. For the comparison of the eucalyptus oil and lemon tea tree oil pediculicide with the "suffocation" pediculicide the formula was Logit(Y) = 1.8651 + (Treatment * 0.1749) + (Time * -2.8370) + (Interaction * 2.5796) whereas for the comparison of the eucalyptus oil and lemon tea tree oil pediculicide with the melaleuca oil and lavender oil pediculicide the formula was Logit(Y) = 2.1182 + (Treatment * -0.0782) + (Time * -2.1334) + (Interaction * 1.8761). The "fixed effects" were the pediculicide, whether the egg was collected pre-treatment or post-treatment, and the interaction of these two "fixed effects", whereas the "random effect" was the subject from whom the eggs were collected. Since the pre-treatment and post-treatment eggs were collected from the same individual subject, the model was a model of repeated measures.

#### Ethics

This trial was conducted in compliance with the World Medical Association Declaration of Helsinki; the requirements of the National Statement on Ethical Conduct in Research Involving Humans and ICH E6 Guidance for the Industry; Good Clinical Practice: Consolidated Guidance; the National Privacy Principles and relevant State/territory laws. The trial activities were approved by the Medical Research Ethics Committee of the University of Queensland and all parents/guardians provided written informed consent.

## Results

### Study population

The treatment groups were not statistically different with regard to gender (p = 0.5603), school attended (p = 0.9811), school grade (p = 0.8170) or hair type (p = 0.8860) (Table 1). Neither did mean hair length differ among the treatment groups (p = 0.8047). But hair type varied from one treatment group to another (p = 0.0308): brown hair was most common in the EO/LTTO treatment group, black hair in the "suffocation" treatment group, whereas brown and blonde were most common in the TTO/LO treatment group (Table 1).

**Table 1:** Subject demographics (%)

Parameter	Value	EO/LTTO (n = 30)	"Suffocation" pediculicide (n = 31)	TTO/LO) (n = 31)	Overall (n = 92)	p
Gender^a^	Female	22 (73.3)	19 (61.3)	22 (71.0)	63 (68.5)	0.5603
	Male	8 (26.7)	12 (38.7)	9 (29.0)	29 (31.5)	
School Code^a^	C	11 (36.7)	11 (35.5)	12 (38.7)	34 (37.0)	0.9811
	D	13 (43.3)	13 (41.9)	14 (45.2)	40 (43.5)	
	RE	6 (20.0)	7 (22.6)	5 (16.1)	18 (19.6)	
Grade^b^	Prep	6 (20.0)	5 (16.1)	8 (25.8)	19 (20.7)	0.8170
	1	10 (33.3)	9 (29.0)	8 (25.8)	27 (29.3)	
	2	1 (3.3)	1 (3.2)	0 (0.0)	2 (2.2)	
	3	4 (13.3)	3 (9.7)	7 (22.6)	14 (15.2)	
	4	4 (13.3)	5 (16.1)	1 (3.2)	10 (10.9)	
	5	2 (6.7)	3 (9.7)	5 (16.1)	10 (10.9)	
	6	2 (6.7)	3 (9.7)	1 (3.2)	6 (6.5)	
	7	1 (3.3)	2 (6.5)	1 (3.2)	4 (4.3)	
Hair Colour^b^	Black	7 (23.3)	16 (51.6)	6 (19.4)	29 (31.5)	0.0308
	Blonde	8 (26.7)	7 (22.6)	12 (38.7)	27 (29.3)	
	Brown	14 (46.7)	6 (19.4)	13 (41.9)	33 (35.9)	
	Red	1 (3.3)	2 (6.5)	0 (0.0)	3 (3.3)	
Hair Length^c ^(cm)	n	30	31	31	92	0.8047
	Mean	28.9	28.8	31.4	29.7	
	SD	15.76	19.63	16.89	17.37	
	Min	3	2	3	2	
	Median	32.0	30.0	33.0	32.0	
	Max	53	59	60	60	
Hair Type^b^	Curly	2 (6.7)	2 (6.5)	1 (3.2)	5 (5.4)	0.8860
	Straight	24 (80.0)	23 (74.2)	23 (74.2)	70 (76.1)	
	Wavy	4 (13.3)	6 (19.4)	7 (22.6)	17 (18.5)	

a: Probability calculated by Pearson's chi-square test; b: Probability calculated by Fisher's Exact test; c: Probability calculated by analysis of variance.

### Efficacy

**Figure 1:**
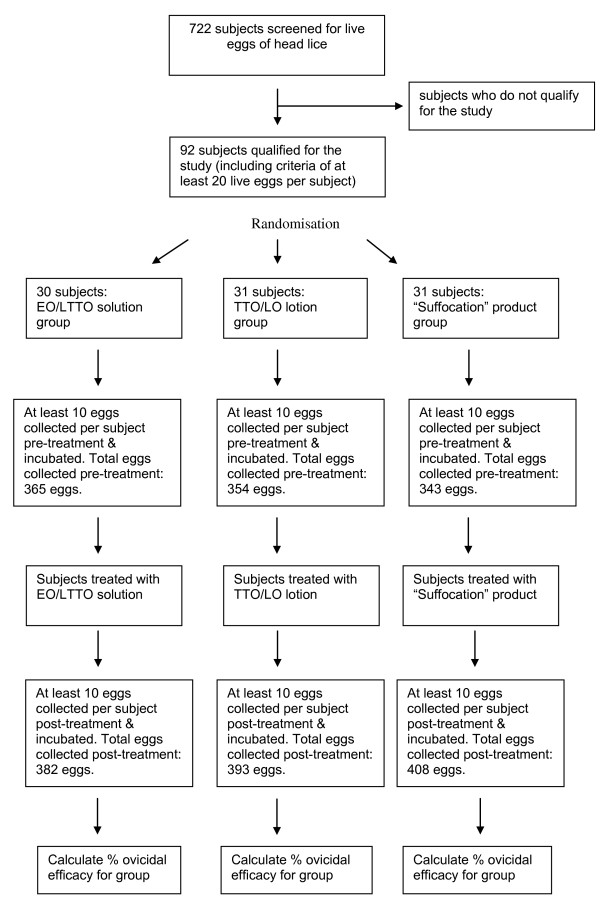
**Disposition of subjects treated with eucalyptus oil and lemon tea tree oil (EO/LTTO), melaleuca oil and lavender oil (TTO/LO), and the suffocation pediculicide**.

722 subjects were screened for live head lice eggs between 9 October 2009 and 7 December 2009. 92 of these subjects were enrolled in the trial: "suffocation" pediculicide (n = 31), TTO/LO (n = 31) and EO/LTTO (n = 30 subjects) (Figure 1). A minimum of 10 eggs and a maximum of 22 eggs were collected from each subject, pre-treatment; whereas a minimum of 10 and maximum of 41 eggs were collected from each subject, post-treatment. All 92 subjects enrolled in the trial completed the trial. There was, however, one protocol deviation: a Petri dish broke and 4 of the 16 eggs collected post-treatment for one subject treated with TTO/LO were lost. Thus, 12 eggs rather than 16 eggs, post-treatment, were examined for hatching for this subject.

#### Results of analyses with the Generalised Estimating Equations (GEE) framework

The *ovicidal efficacy *of the EO/LTTO was 3.3% (SD 16%) whereas the *ovicidal efficacy *of the TTO/LO was 44.4% (SD 23%) and the *ovicidal efficacy *of the "suffocation" pediculicide was 68.3% (SD 38%). The *ovicidal efficacies *of EO/LTTO and TTO/LO were statistically different (p < 0.0001) (Table 2). The *ovicidal efficacies *of EO/LTTO and the "suffocation" pediculicide were also statistically different (p < 0.0001) (Table 2).

**Table 2:** Cumulative number (No

Treatment	Pre-treatment OR post-treatment	No. Eggs collected	No. Eggs hatched	Hatch- rate	% OE	p*
"Suffocation" pediculicide	Pre-	343	297	0.8659		
	Post-	408	112	0.2745	68.3%	< 0.0001
Melaleuca oil & lavender oil (TTO/LO)	Pre-	354	316	0.8927		
	Post-	393	195	0.4962	44.4%	< 0.0001
Eucalyptus oil & lemon tea tree oil (EO/LTTO)	Pre-	365	323	0.8849	3.3%	
	Post-	382	327	0.8560		

p*-values are for comparisons of "suffocation' pediculicide and TTO/LO Lotion with EO/LTTO Solution.

#### Comparison of ovicidal efficacies in the subjects treated with EO/LTTO and the "suffocation" pediculicide

Hair type, hair colour, subject gender and school attended did not co-vary with *ovicidal efficacy *in the analysis of subjects treated with EO/LTTO and the "suffocation" pediculicide. Hair length, however, co-varied with *ovicidal efficacy *in this analysis (p = 0.0471): both treatments were less effective on longer hair compared to shorter hair. Also, both treatments were less effective on subjects from grades 3 (p = 0.0020), 4 (p = 0.0198) and 7 (p = 0.0173) than the other grades.

#### Comparison of ovicidal efficacies in the subjects treated with EO/LTTO and TTO/LO

Hair type, hair colour, gender and hair length did not co-vary with *ovicidal efficacy *in the analysis of subjects treated with EO/LTTO and TTO/LO. The *ovicidal efficacy *of both treatments in this analysis was, however, lower in one of the three schools attended compared to the other two schools attended (p = 0.0033).

### Safety (tolerance)

There were 15 Adverse Events (AEs). One of these AEs was judged by the Investigator to be "possibly" related to the study treatment whereas all the others were considered "probably" related to the study treatment due to the time course of the events. Six of 30 subjects (20%) treated with EO/LTTO reported an AE whereas 4 of 31 (12.9%) treated with TTO/LO reported an AE; no AEs were reported in the "suffocation" pediculicide treatment group. These differences were statistically significant (Fisher's Exact test p = 0.0026). All AEs were graded by the Investigator as mild in severity (rating scale: mild, moderate or severe) and there were no serious AEs. No action was required for any AEs and all resolved.

Two types of adverse events occurred in the EO/LTTO and TTO/LO treatment groups: redness and stinging. All AEs involved redness. The average duration of redness following treatment with EO/LTTO was 28.5 minutes compared to 53.8 minutes following treatment with TTO/LO. Stinging was reported by 2 subjects who were treated with EO/LTTO (2/30 or 6.7%) and by 3 subjects treated with TTO/LO (3/31 or 9.7%). The average duration of stinging following EO/LTTO was 10 minutes compared to 24 minutes following TTO/LO.

## Discussion

Neither hair type nor hair colour co-varied with ovicidal efficacy in our study, despite a range of ethnic groups in the study population. Therefore data from *ex vivo *studies like ours in Brisbane should be indicative of efficacy in other populations in Australia i.e. be generalisable.

Ideally, pediculicides are both lousicidal (kill lice) and ovicidal (kill un-hatched nymphs in the eggs). Dodd [2] argued, that the most accurate way to compare the ovicidal efficacy of pediculicides is to collect eggs pre-treatment from infected individuals, treat those individuals with a pediculicide, and then collect eggs from the same infected individuals, post-treatment. Thus, the proportion of eggs pre-treatment and post-treatment that hatch can then be compared precisely (hatch-rate), for each individual subject and for each pediculicide. This study design has the advantage of using the individual subject as his/her own control. However, as Sonnberg et al pointed out, the age of the eggs on the head at time of the test are not known and rearing conditions are not standardized [6]. It will be interesting to see whether or not the ovicidal efficacy differs between tests where the eggs are treated on the scalp [our study, 3,4,5,7 & 8] compared to tests where the eggs were grown *ex vivo *off the scalp by the continuous feeding method (ie in a Plexiglas receptacle attached to the wrist of volunteers) [6,9]. "League tables" of clinical efficacy for each country/region might be generated by these approaches. "League tables" like these would help consumers choose pediculicides and might encourage pharmaceutical companies to make more efficacious pediculicides.

## Conclusions

Ovicidal efficacy varied substantially among treatments, from 3.3% to 68.3%. We conclude that the "suffocation" pediculicide and TTO/LO had significant ovicidal activity (killed 68.3% and 44.4%, respectively, of live head lice eggs) following a single application to the scalp and hair according to the manufacturers' instructions whereas EO/LTTO had virtually no ovicidal activity (3.3%) (p < 0.0001). The "suffocation" pediculicide and TTO/LO are also highly efficacious against the crawling stages [1]. Thus our ranking: 1) "Suffocation pediculicide" (68.3% efficacy against eggs); 2) TTO/LO (44.4% efficacy against eggs) 3) EO/LTTO (3.3% efficacy against eggs). The "suffocation" pediculicide and TTO/LO should be recommended as first line treatments.

## Competing interests

Stephen Barker and Dr. Phillip Altman have previously executed clinical trials for, and provided consultant advice to, Key Pharmaceuticals Pty. Ltd., the company that funded this study. The company did not have an active role in the study design, study management, data analysis or interpretation of results. Company representatives did, however, read a draft of the manuscript and make suggestions, some of which were accepted by the authors.

## Authors' contributions

PMA and SCB designed the study. PMA drafted the protocol and the report, supervised site-staff training, managed the study and monitored the study. SCB conducted the trial and wrote the manuscript. PMA read and approved the final manuscript.

## Pre-publication history

The pre-publication history for this paper can be accessed here:

http://www.biomedcentral.com/1471-5945/11/14/prepub
